# Co-Occurrence of Interleukin-6 Receptor Asp358Ala Variant and High Plasma Levels of IL-6: An Evidence of IL-6 Trans-Signaling Activation in Deep Vein Thrombosis (DVT) Patients

**DOI:** 10.3390/biom12050681

**Published:** 2022-05-10

**Authors:** Rossella Salemi, Giuseppe Gattuso, Barbara Tomasello, Alessandro Lavoro, Agostino Gaudio, Massimo Libra, Salvatore Santo Signorelli, Saverio Candido

**Affiliations:** 1 Department of Biomedical and Biotechnological Sciences, University of Catania, 95123 Catania, Italy; rossellasalemi2580@gmail.com (R.S.); peppeg9305@gmail.com (G.G.); alessandrolavoro@ymail.com (A.L.); mlibra@unict.it (M.L.); 2 Department of Drug and Health Sciences, University of Catania, 95123 Catania, Italy; btomase@unict.it; 3 Department of Clinical and Experimental Medicine, University of Catania, 95123 Catania, Italy; agostino.gaudio@gmail.com (A.G.); ssignore@unict.it (S.S.S.); 4 Internal Medicine Division, University Hospital ‘G. Rodolico’, 95123 Catania, Italy; 5 Research Center for Prevention, Diagnosis and Treatment of Cancer, University of Catania, 95123 Catania, Italy

**Keywords:** interleukin-6, interleukin-6-receptor, trans-signaling, inflammation, deep vein thrombosis

## Abstract

Interleukin-6 (IL-6) is a pleiotropic cytokine involved in several mechanisms, and the alteration of IL-6 signaling leads to the overactivation of various processes including immunity, inflammation, and hemostasis. Although IL-6 increase has been documented in venous thromboembolic diseases, the exact involvement of IL-6 signaling in deep vein thrombosis (DVT) has not been fully understood. Consequently, we investigated the involvement of IL-6 trans-signaling in inflammatory events occurring in DVT, focusing on the role of the interleukin-6 receptor (IL6-R) Asp358Ala variant. The circulating levels of IL-6, soluble IL6-R (sIL6-R), and soluble glycoprotein 130, as well as the Asp358Ala genotyping, were assessed in a consecutive cohort of DVT patients and healthy controls. The results indicated that IL-6 was higher in DVT compared to controls. Moreover, sIL6-R levels were strongly correlated to Asp358Ala variant in both groups, showing a high frequency of this mutation across all samples. Interestingly, our results showed a high frequency of both Asp358Ala mutation and raised IL-6 levels in DVT patients (OR = 21.32; *p* ≤ 0.01), highlighting that this mutation could explain the association between IL-6 overactivation and DVT outcome. Overall, this study represents a proof of concept for the targeting of IL-6 trans-signaling as a new strategy for the DVT adjuvant therapy.

## 1. Introduction

Interleukin-6 (IL-6) displays pleiotropic activities orchestrating multiple processes in the body. Specifically, IL-6 plays a key role in metabolic disorders (e.g., lipid metabolism and insulin resistance), neuroendocrine diseases, neuropsychological behavior, mitochondrial activities, and vascular diseases [1,2,3,4,5,6,7]. IL-6 also promotes the clonal expansion, T-cell activation, the differentiation of B cells, and the regulation of the acute-phase response [8,9].

A paradoxical effect has been deeply described for IL-6 that plays a protective role modulating the innate and adaptive immunity and meanwhile induces harmful effects stimulating inflammatory processes in chronic inflammation-related diseases (e.g., systemic lupus, plasmacytoma, autoimmune encephalomyelitis, and chronic arthritis) as well as cancer [4,10,11].

It has been demonstrated that single-nucleotide polymorphisms (SNPs) in the *IL-6* gene may modify the plasma levels and biological activity of this pro-inflammatory cytokine, affecting the outcome of inflammatory diseases [12,13,14]. Some of these SNPs are located in the *IL-6* promoter region, leading to transcriptional overexpression [15,16,17]. Furthermore, it has been recently demonstrated that some SNPs were associated with IL-6 trans-signaling occurring in chronic inflammatory diseases [2,18]. 

It is well known that IL-6 trans-signaling is activated by the interaction between IL-6 and the soluble form of IL-6 receptor (sIL-6R) derived from expression of IL-6R isoform lacking membrane domain or proteolytic cleavage of anchored receptor by a disintegrin and metalloproteases (ADAMs) enzymes. Notably, this cleavage is strongly enhanced by Asp358Ala mutation occurring in the extracellular domain of IL-6R [19,20,21]. The interaction between IL-6 and sIL-6R raises the circulating half-life of IL-6 and promotes its biological effects [22]. Additionally, the IL-6:sIL-6R complex is enable to bind glycoprotein 130 (gp130) widely expressed on the surface of several cell types, which as consequence become responsible for IL-6 when they are normally unaffected by this cytokine. On the other hand, the soluble gp130 (sgp130) binding the IL-6:sIL-6R complex prevents its interaction with gp130 on the cell surface and the activation of IL-6 trans-signaling in IL-6 unresponsive cells [19]. Similar to IL-6R, the sgp130 secretion depends on the expression of spliced IL-6 signal transducer (IL6ST) mRNA lacking the transmembrane domain [23]. Despite sgp130 representing an important physiological buffer of IL-6 trans-signaling attenuating the IL-6 overactivation, sgp130 may be inadequate to counteract IL-6 trans-signaling during inflammation, in which IL-6 and sIL-6R levels are strongly increased [23,24].

In the last decades, the relationship between deep vein thrombosis (DVT) and inflammation has been widely investigated, focusing on the role of IL-6 [25,26,27]. In particular, IL-6 seems to play a critical role in inflammation-related thrombosis, together with tumor necrosis factor-alpha (TNFα), interleukin 8 (IL-8), and C-reactive protein (CRP), representing powerful risk predictors for DVT [28]. It was demonstrated that these inflammatory molecules are involved in venous thromboembolism (VTE) and DVT by promoting the pro-coagulant condition by inducing the expression of tissue factor (TF) [29]. 

Despite several pieces of scientific evidence strongly highlighting the involvement of IL-6 in DVT, the complex interplay among IL-6 pathway, inflammation, and DVT is not fully understood. To the best of our knowledge, the role of IL6R and IL6ST expression and post-translational modifications on IL-6 response in DVT development has been poorly investigated [19].

On these bases, the plasma levels of IL-6, sIL-6R, and sgp130 as well as the rs2228145 SNP were assessed in a cohort of 19 DVT patients and 22 healthy controls to evaluate the role of IL-6 cis- and trans-signaling in DVT.

## 2. Materials and Methods

### 2.1. Patients and Healthy Controls

A consecutive cohort of 19 patients with DVT of the lower limbs (median age range: 46–71 years) and 22 healthy subjects (median age range: 41.7–62 years) were enrolled at the Internal Medicine Department of G. Rodolico University Hospital (Catania, Italy). The ultrasound (US) examination of the venous circulation of the lower limbs was used for DVT diagnosis. Specifically, the non-compressibility of the vascular lumen by the US probe was settled as an inclusion diagnostic criterion of lower limb DVT. Patients with provoking risk factors including pulmonary embolism, genetic thrombophilia, trauma and surgery, inflammatory bowel disease, liver disorder, malignancy, and pregnancy were deemed ineligible. Blood samples were collected at the moment of DVT diagnosis to obtain serum, plasma, and buffy coat.

The healthy individuals were enrolled at the Noninvasive Vascular Laboratory of G. Rodolico University Hospital (Catania, Italy). The subjects suffering from diseases including thrombosis, cardiovascular disorders, malignancies, rheumatic and inflammatory bowel diseases, and chronic renal or liver diseases were excluded from study. Both patients and healthy individuals were not treated with any medication such as anticoagulant and anti-inflammatory drugs as well as dietary supplements.

The patients and controls were from the same geographic region and had similar ethnic background and were matched for age (>18 yeas). The study was conducted in accordance with the Declaration of Helsinki, and the protocol was approved by the Ethics Committee of the Garibaldi Hospital (Catania, Italy; resolution n.23/2016/CECT2). Both patients and controls were informed about the research and were asked to give their written consent. 

The demographic and clinical characteristics of all the patients and controls are reported in Table 1.

### 2.2. Sample Collection and ELISA Test

A total of 5 mL of venous blood was collected in EDTA vacuum tubes, centrifuged at 2000× *g* for 10 min at room temperature to separate the plasma and buffy coat fractions, and then stored at −80 °C until use.

IL-6, sIL-6R, and sgp130 plasma levels were detected in triplicate by enzyme-linked immunosorbent assay using commercially available kits (Human IL-6 HS-HS600C, Lot 314515; Human IL-6 Rα-DR600, Lot P226142; Human soluble gp130-DGP00, Lot P199893; Quantikine, R&D system, Minneapolis, MN, USA), according to the manufacturer’s protocol.

The Inter-Assay CV and the Intra-Assay CV of each ELISA test were evaluated to assess the assay precision and the repeatability. The inter-assay CVs (n = 2 plates) (average of high and low control CV) were IL-6 HS, 3,36%; IL-6 Rα, 3.02%; soluble gp130, 3.7%. Moreover, the intra-assay CVs (average % CV) for DVT patients were IL-6 HS, 3,80%; IL-6 Rα, 3.82%; soluble gp130, 2.23%. For healthy controls, the intra-assay CVs (average % CV) were IL-6 HS, 5,10%; IL-6 Rα, 4.54%; soluble gp130, 1.90%.

### 2.3. IL6R Exon 9 Sequencing

Genomic DNA was extracted from the buffy coat collected from patients and controls using the standard phenol-chloroform method. IL6R exon 9 sequence (chr1:154,426,802–154,427,232-GRCh37/hg19) was amplified by PCR using the DreamTaq™ Hot Start PCR Master Mix (Cat. N. K9011-Thermo Fisher Scientific™, Waltham, MA, USA). The primers and amplification conditions were as follows: Fw: 5′-TGTTGGTTGGCAGAGCTGTT-3′ and Rev: 5′-CACCTAAAACACGGCTTGGC-3′; 1 cycle of 94 °C for 3 min, followed by 35 cycles of 94 °C for 30 s, 60 °C for 30 s, and 72 °C for 90 s, followed by 1 cycle of 72 °C for 10 min.

The PCR product was purified with the GeneJET PCR Purification Kit (Cat. No. K0702-Thermo Fisher Scientific™, Waltham, MA USA) and sequenced with the Mix2Seq Kits (Eurofins Genomics, Germany GmbH, Ebersberg, Germany) according to the manufacturer’s instructions. The obtained sequences were compared to the NCBI reference sequences to identify any polymorphic variants within the Exon 9 of the *IL6R* gene. The analysis of DNA sequences was performed by Chromas Lite software version 2.6.6 (accessed on 18 January 2022—technelysium.com.au/wp/) (Technelysium Pty Ltd., South Brisbane, Brisbane, Australia).

### 2.4. Statistical Analysis

The Mann–Whitney test was performed for differential analyses between the groups of patients and healthy controls. Normality of data was assessed using the Shapiro–Wilk test. Comparisons between categorical variables were performed by Fisher’s exact test. The statistical test was performed using GraphPad Prism software (Version 8.0.2) (GraphPad Software, San Diego, CA, USA). All analyses were performed in triplicate, and *p*-value ≤ 0.05 was considered statistically significant.

## 3. Results

### 3.1. Demographic and Clinical Characteristics of Patients and Healthy Controls

A summary of the basic characteristics of the enrolled subjects is reported in Table 1. The median ages of the patients and controls were 56 (46–71) and 48 (41.7–62), respectively (*p* = 0.239). There was no significant difference between patients and controls considering gender distribution (*p* = 0.76).

### 3.2. Plasma Levels of IL-6, sIL-6R, and sgp130

**Figure 1:**
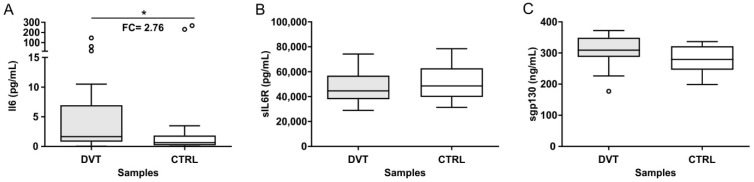
Plasmatic levels of IL-6, sIL-6, and sgp130 in DVT patients and healthy controls. (**A**) Box plot representation of the IL-6 levels in DVT patients compared to healthy controls (* *p* ≤ 0.05). (**B**) Box plots representation of the sIL-6R levels in DVT patients compared to healthy controls. (**C**) Box plots representation of the sgp130 levels in DVT patients compared to healthy controls.

The ELISA analysis showed that the IL-6 plasma levels were significantly (*p* ≤ 0.05) higher in DVT patients (1.57 pg/mL, range 0.73–5.98 pg/mL) compared to healthy controls (0.57 pg/mL, range 0.17–1.47 pg/mL) (Figure 1A). Conversely, no statistically significance was observed when analyzing the plasmatic levels of sIL-6R and sgp130 in both comparing groups (Figure 1B,C).

**Figure 2:**
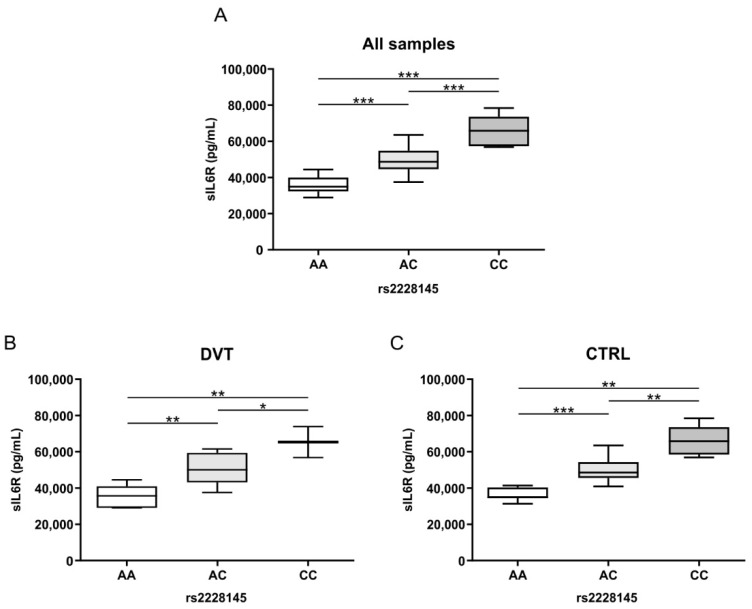
Distribution of sIL-6R levels according to IL6R rs2228145 SNP. (**A**) Box plots of sIL-6R levels (pg/mL) for all samples stratified according to SNP rs2228145 genotype (AA, AC, CC) (*** *p* ≤ 0.001). (**B**) Box plots of sIL-6R levels (pg/mL) for DVT patients stratified according to SNP rs2228145 genotype (AA, AC, CC) (* *p* ≤ 0.05; ** *p* ≤ 0.01). (**C**) Box plots of sIL-6R levels (pg/mL) for healthy controls stratified according to SNP rs2228145 genotype (AA, AC, CC) (** *p* ≤ 0.01; *** *p* ≤ 0.001).

Figure 2A shows that sIL-6R plasma levels were higher in subjects (DVT and healthy control) harboring the AC and CC rs2228145 SNPs compared to the wild type (AA) groups (AA: 34,993 pg/mL, range 32,333–39,999 pg/mL; AC: 48,728 pg/mL, range 44,633–54,808 pg/mL; CC: 65,916 pg/mL, range 57,416–73,629 pg/mL–*p* ≤ 0.001). A similar trend was observed for sIL6R serum levels in DVT patients (AA: 35,612 pg/mL, range 29,018–40,805 pg/mL; AC: 50,095 pg/mL, range 43,123–59,511 pg/mL; CC: 65,512 pg/mL, range 56,851–74,173 pg/mL) (Figure 2B) and healthy controls (AA: 34,993 pg/mL, range 34,468–40,397 pg/mL; AC: 48,503 pg/mL, range 45,651–54,263 pg/mL; CC: 65,916 pg/mL, range 58,429–73,609 pg/mL) (Figure 2C). In all cases the sIL6R serum levels were significantly higher (*p* ≤ 0.05) in the presence of rs2228145 A > C substitution, and the highest levels in particular were observed for the CC homozygous samples (Figure 2).

### 3.3. Association Analysis of IL-6 Plasma Levels and IL6R Polymorphisms in DVT

Fisher’s exact test was used to verify the association between the IL-6 plasma levels and the rs2228145 SNP status of sIL6R in the DVT patients and healthy subjects. 

The results showed a moderate association (OR 5.7, *p* ≤ 0.05) of raised IL-6 plasma levels with DVT, while no association was found between DVT and rs2228145 SNP. We postulate that this result was due to the uniform distribution of IL6R rs2228145 SNP among the DVT patients and controls (Table 2, Figure S1). 

Conversely, a high frequency of Asp358Ala variant was found in DVT patients with IL-6 levels higher than 3.465 pg/mL (i.e., the 75th percentile of IL-6 values in normal subjects) (DVT = 33.33%, control = 0%; OR > 20) (Table 2).

## 4. Discussion

Over the years, an increasing number of studies have focused on defining new therapeutic strategies to target IL-6, its receptors, or signaling pathway [2,30]. The research was inspired by the evidence that IL-6 is a key cytokine supporting chronic inflammatory diseases and the potential progression of their outcome (e.g., arthritis and other immune-system mediated diseases) [4]. Although it is known that IL-6 plays a protective role in defense against infections, it can also be involved in maintaining chronic inflammation [31]. A variety of dynamic downstream events occur after the stimulation of IL-6 signal transduction due to the activation of either the cis- or trans-signaling, which mainly depends on the post-transcriptional and post-translational modifications of IL-6R and gp130 membranous proteins [19]. IL-6 cis-signaling is physiologically active in cells that normally express IL6-R and gp130 on their surface [2]. On the other hand, trans-signaling occurs when cells release sIL-6R by proteolytic cleavage or expressing IL6-R isoform lacking transmembrane domain. sIL-6R may bind IL-6, resulting in both IL-6 half-life increase and IL-6 responsiveness of cells normally refractory. Of note, these cells do not express IL-6R under physiological conditions, while gp130 is ubiquitously synthetized. Mechanistically, sIL-6R:IL-6 complex may be recruited on the surface of these cells by the gp130 protein activating the underlying signal cascade of IL-6 [19,20,21,22]. A number of studies have reported high levels of IL-6 in patients suffering from DVT as well as IL-6 levels lowering during direct oral anticoagulant and heparin administration, thus demonstrating the anti-inflammatory properties of heparin, one of the most largely used anti-thrombotic drugs [32,33]. This last knowledge reinforces the central role of inflammatory cytokines such as IL-6 in the pathophysiological mechanism involved in DVT [26,28,34]. 

In this study, we observed raised IL-6 plasma levels in DVT patients compared to controls. Similarly, CRP was found to be higher in DVT patients compared to controls. This evidence adds proof to identify IL-6 as an inflammatory agent able to promote thrombotic events in deep veins (i.e., the DVT). Nevertheless, further analyses are needed to clarify the complex interaction between inflammation and venous thromboembolic diseases. Furthermore, it would be interesting to identify how far IL-6 signaling is active in DVT patients and understand the influence of pleiotropic IL-6 on the potential outcome of thrombotic disease. To define the role played by IL-6 in the pathophysiology of DVT it should be mandatory to investigate the IL-6 trans-signaling involvement. 

Studies have shown that rs2228145 SNP within *IL6R* exon 9 (extracellular domain) is strongly associated with increased levels of the sIL-6R [18,20,21]. In this study was evaluated the role of the *IL-6R* Asp358Ala variant in the increase in sIL-6R plasma levels in DVT and healthy subjects. Furthermore, the plasmatic levels of sgp130 was evaluated to assess its potential effect as decoy receptor of IL-6 in DVT.

The SNP genotyping analysis showed that rs2228145 SNP was identified in 66% of samples showing an equal distribution between patients and controls. This observation suggests that the Asp358Ala mutation is rather common, as previously reported by Reich et al. [35]. Notably, it has been demonstrated that the *IL6R* Asp358Ala variant correlates to increased cleavage of membranous IL-6R protein by specific ADAMs resulting in strong IL-6 trans activation [36,37]. As expected, the sIL-6R plasma levels were significantly higher in samples harboring the A > C variant of rs2228145 SNP compared to the wild type. This result agrees with findings from other studies highlighting the association between this polymorphism and inflammatory activity [38,39,40]. Unlike sIL-6R, the sgp130 plasma levels did not show significant changes in both controls and patients demonstrating that it does not play a significant role in systemic IL-6 trans-signaling activation in DVT patients. We speculate that the buffering role of sgp130 on IL-6 trans-signaling is overcome by increased systemic levels of IL-6 and sIL-6R in inflammatory diseases. 

The contingent analysis revealed that DVT patients exhibited a concomitant occurrence of highly circulating IL6 levels and the Asp358Ala SNP. Accordingly, we postulate that the Asp358Ala variant triggers the overactivation of inflammatory response, exacerbating the IL-6 effect in DVT.

This study can be considered a proof of concept for the involvement of IL-6 signaling alteration in DVT due to a small number of patients that met the exclusion criteria, especially the administration of anticoagulant and anti-inflammatory drugs. Future analyses will be performed on a larger cohort of patients to confirm the involvement of IL-6 trans-signaling in DVT.

## 5. Conclusions

The results of our study provide an interesting point of view about the key role of IL-6 pathway in inflammatory events occurring in DVT patients. This finding may allow for the identification of a subset of DVT patients that may take advantage from selective targeting of IL-6 trans-signaling as an effective therapeutic adjuvant option.

## Figures and Tables

**Table 1:** Demographic and clinical characteristics.

	CTRL	DVT	*p*-Value
Age (years), median (range)	48 (41.7–62)	56 (46–71)	0.2392 *
Gender, number (%)			
Male	12 (54.5)	12 (63.2)	0.76 ^†^
Female	10 (45.5)	7 (36.8)	
CRP, median (range)	2.062 (0.79–6.96)	6 (3–11)	0.04 *

Abbreviations: CRP: C-reactive protein; CTRL: controls; DVT: deep vein thrombosis; *: Mann–Whitney test; ^†^: Fisher’s exact test.

**Table 2:** Contingency analysis according to IL-6 plasma levels and *IL6R* rs2228145 status.

Parameter	DVT Patients Number (%)	Healthy Controls Number (%)	OR, CI *p*-Value
Plasma IL-6			
≥3.465	9 (50)	3 (15)	5.7, 1.22–26.34 ≤0.05
<3.465	9 (50)	17 (85)	
*IL6R* rs2228145 genotype			
AC and CC	13 (68.42)	14 (66.67)	1.08, 0.29–4.08 1.00
Wild type	6 (31.58)	7 (33.33)	
IL-6 and *IL6R* rs2228145 genotype			
≥3.465 and AC and CC	6 (33.33)	0	21.32, 1.10–412.20 ≤0.01
≥3.465 and WT; <3.465 and WT, AC, CC	12 (66.67)	20 (100)	

IL-6 plasma levels were undetectable for 1 DVT patient and 2 healthy controls. *IL6R* rs2228145 SNP status was undetectable for 1 healthy control. Statistical significance was assessed by Fisher’s exact test. Abbreviations: CI: 95% confidence interval; DVT: deep vein thrombosis; OR: odds ratio.

## Data Availability

Not applicable.

## Supplementary Materials

The following supporting information can be downloaded at https://www.mdpi.com/article/10.3390/biom12050681/s1, Figure S1: Representative sequencing chromatograms of the AA, AC, and CC genotypes of *IL6R* rs2228145 SNP.
