# Supplementary figures and images for: Co-Occurrence of Interleukin-6 Receptor Asp358Ala Variant and High Plasma Levels of IL-6: An Evidence of IL-6 Trans-Signaling Activation in Deep Vein Thrombosis (DVT) Patients
Source: Biomolecules. 2022 May 10;12(5):681. doi: 10.3390/biom12050681 (PMC9138210; doi:10.3390/biom12050681)

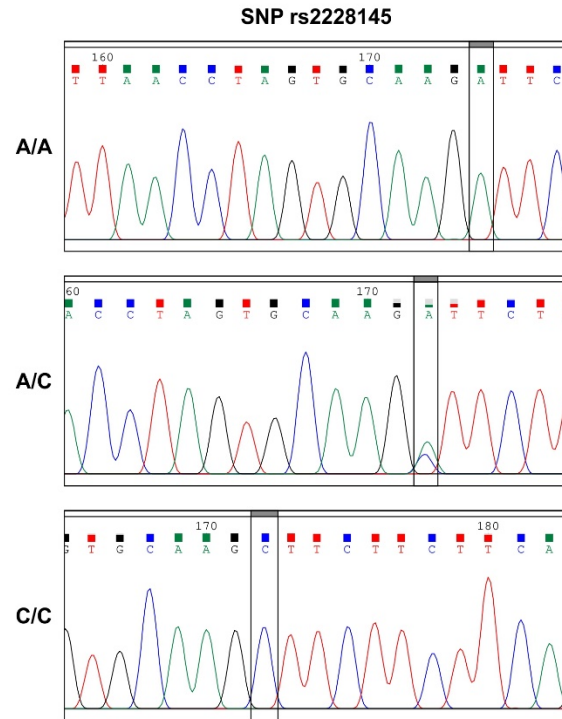

Figure S1. Representative sequencing chromatograms of the AA, AC, and CC genotypes of IL6R rs2228145 SNP.

Supplement: Supplementary file 1 [file biomolecules-12-00681-s001.zip › biomolecules-1702187-supplementary.pdf]
